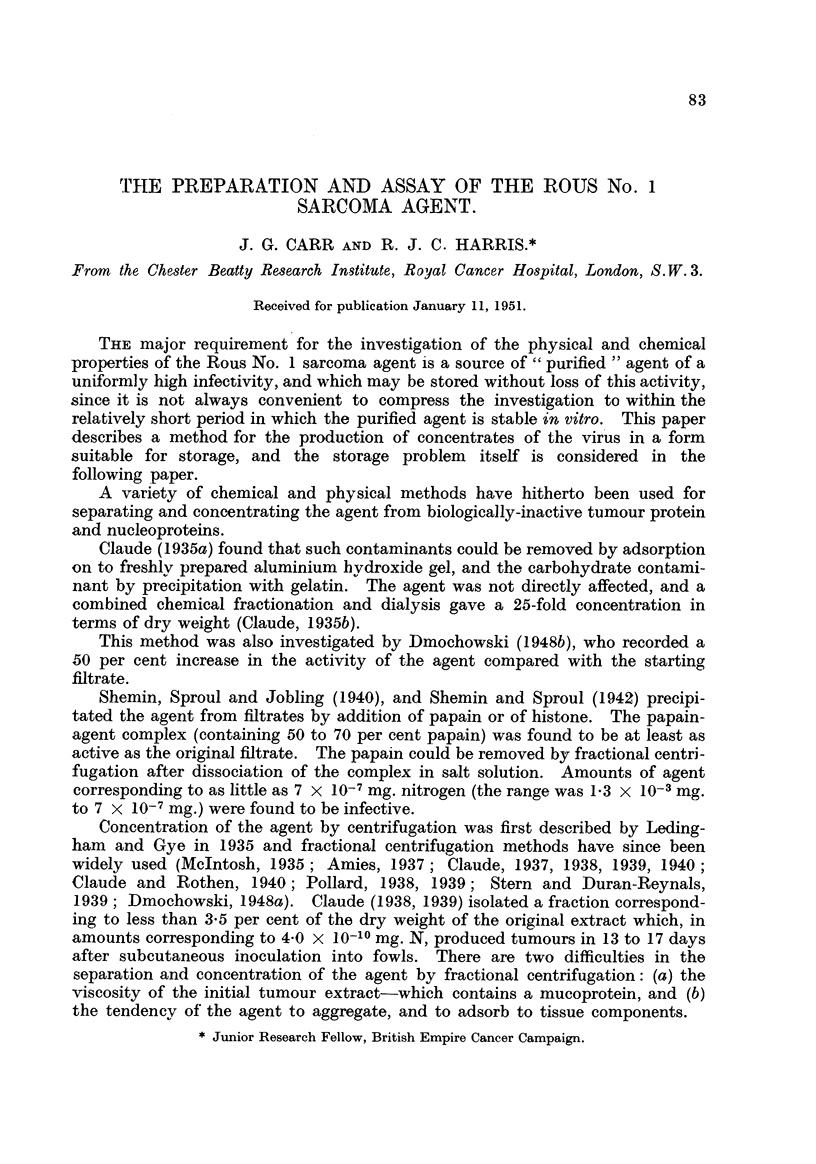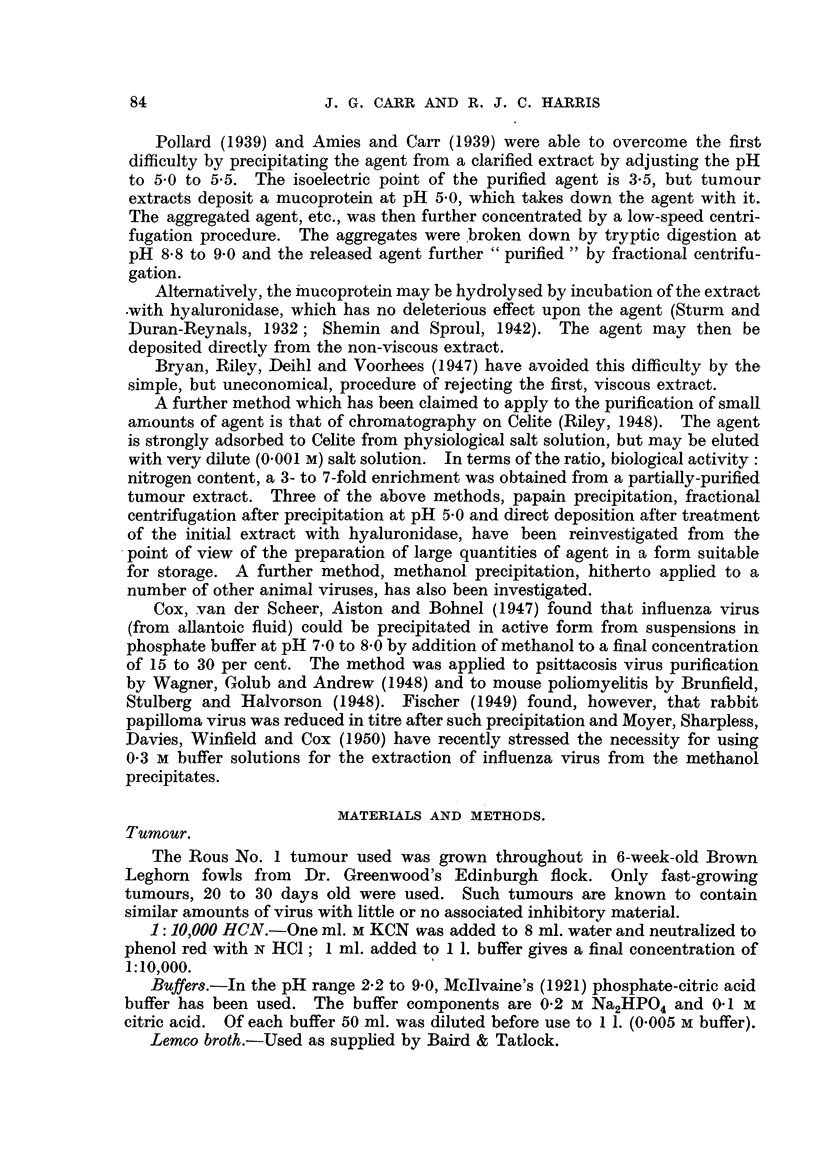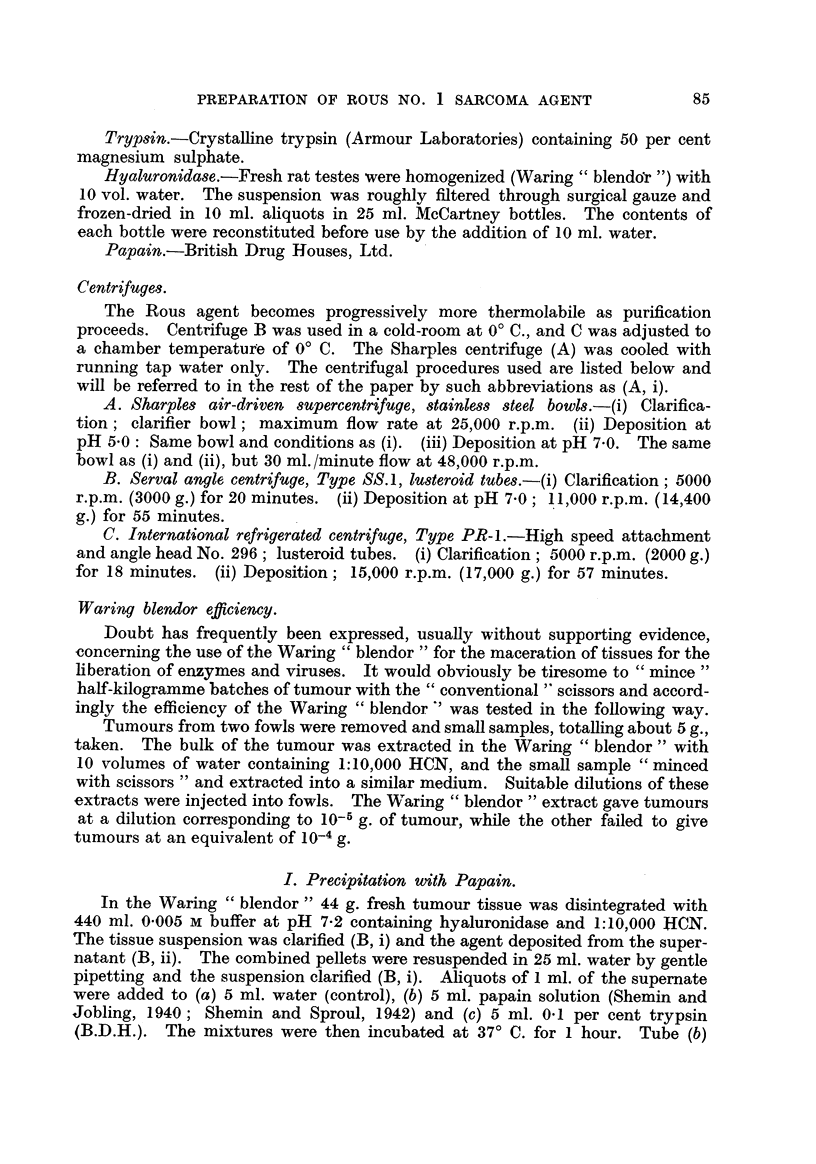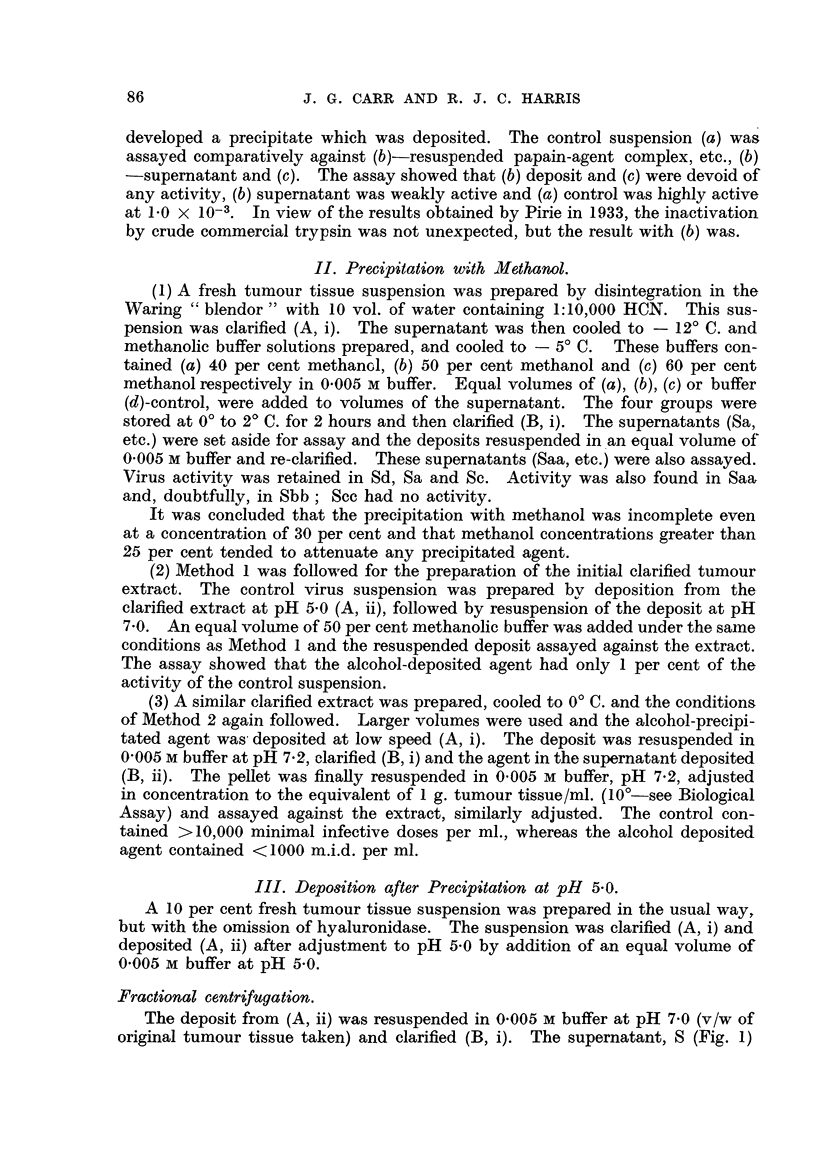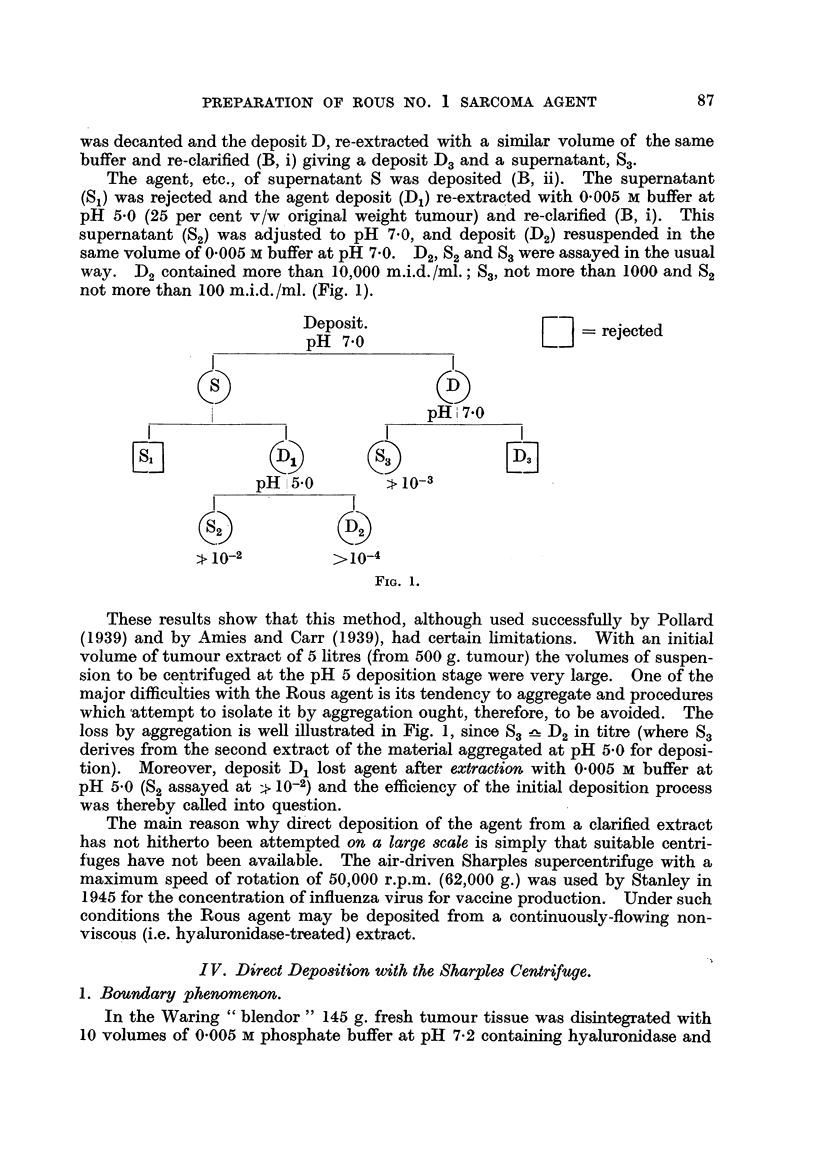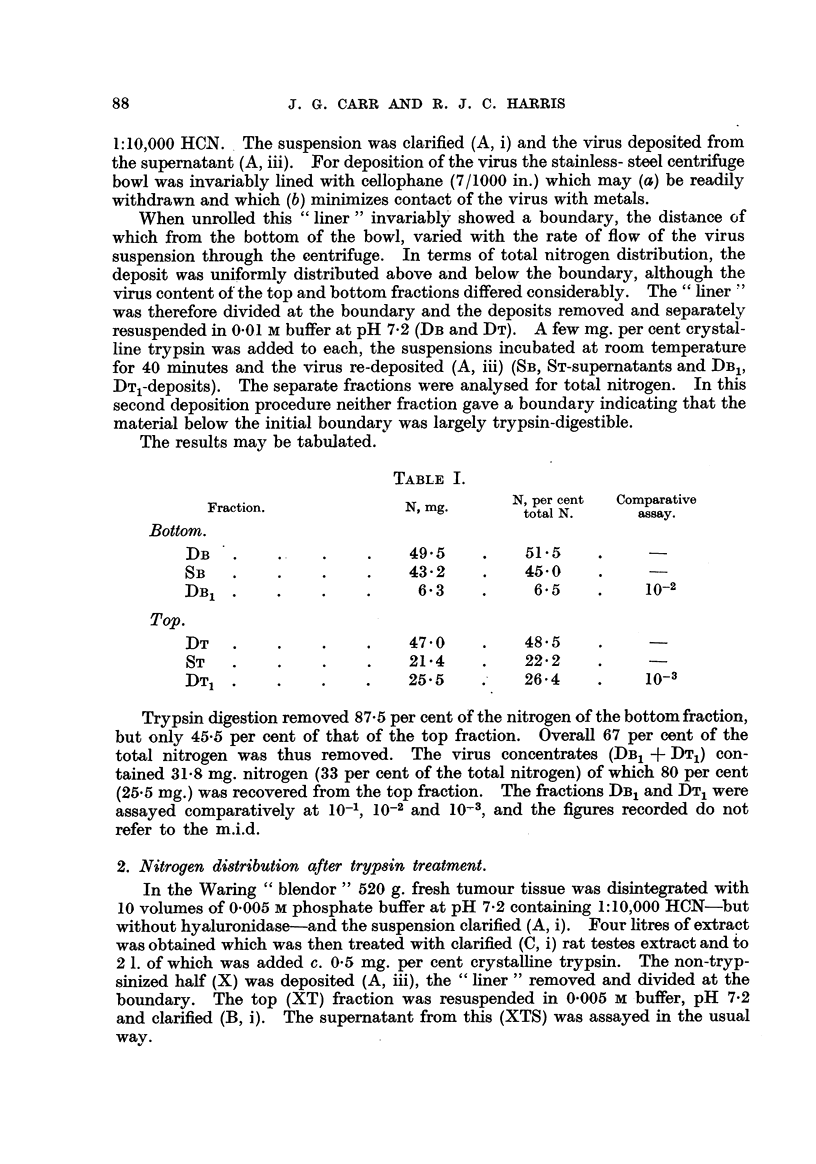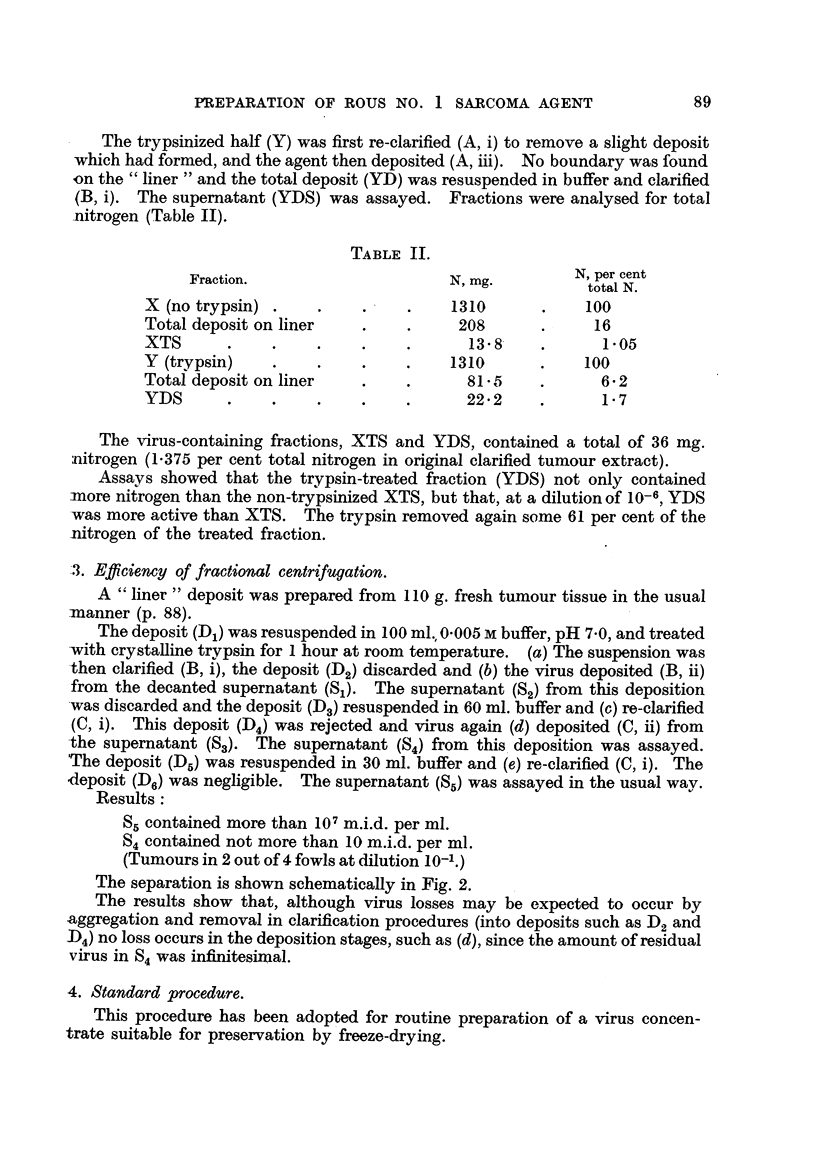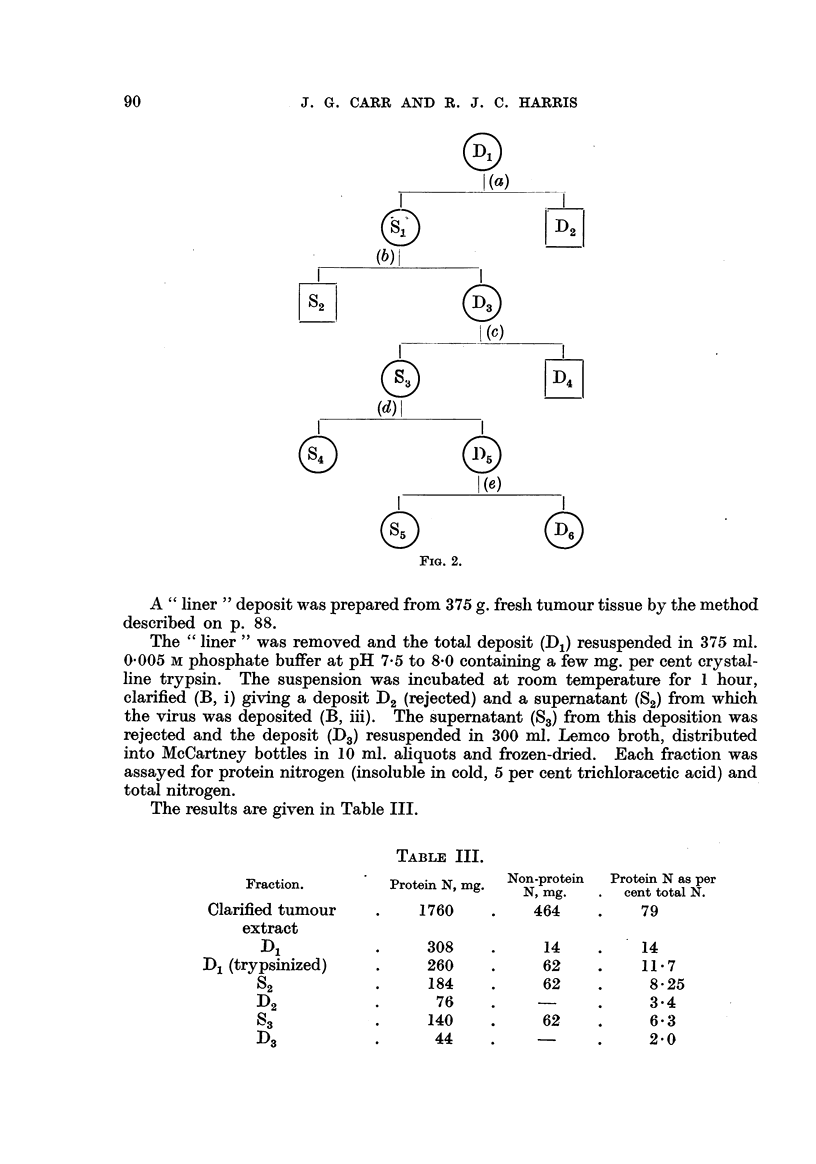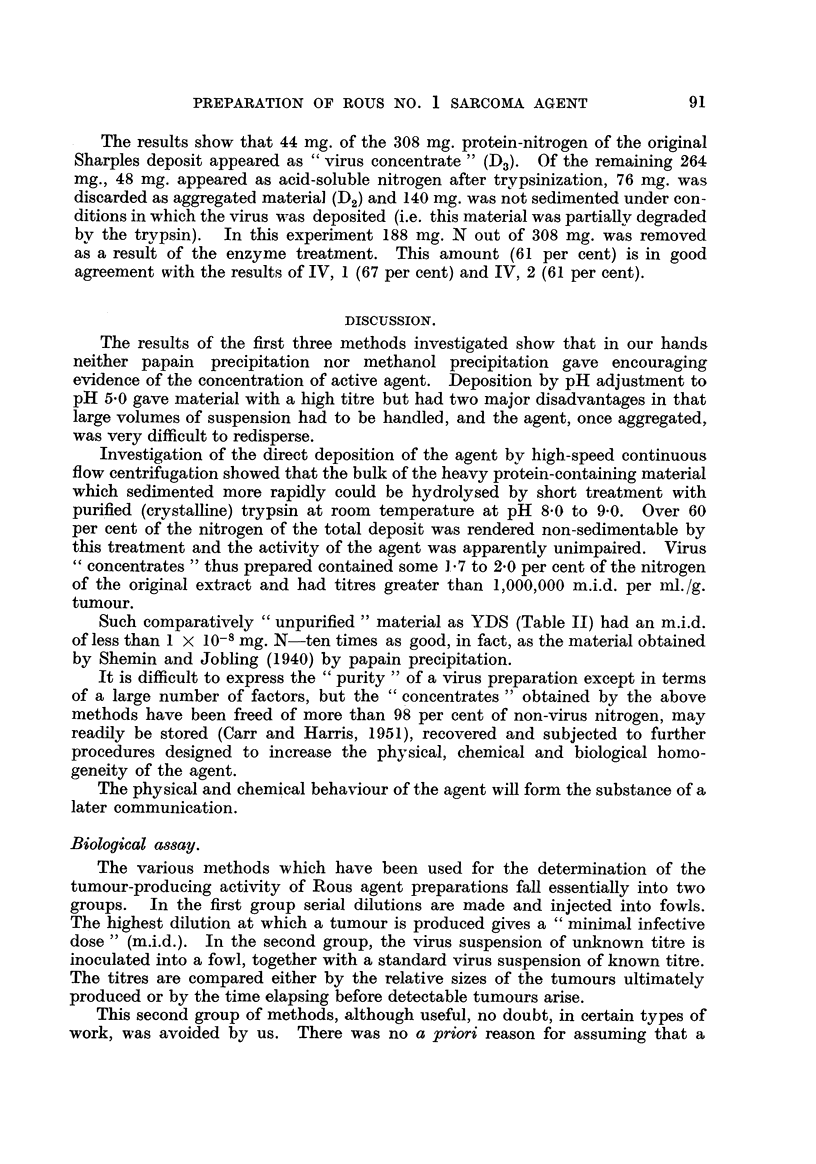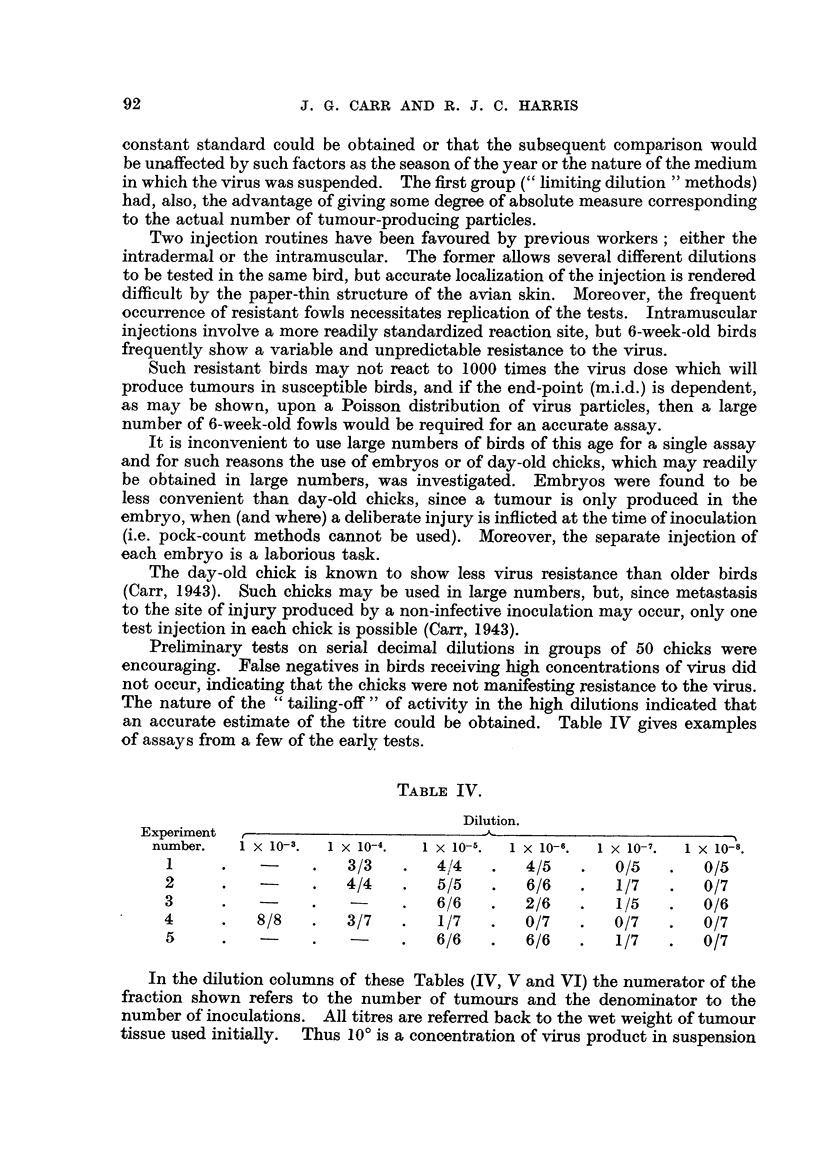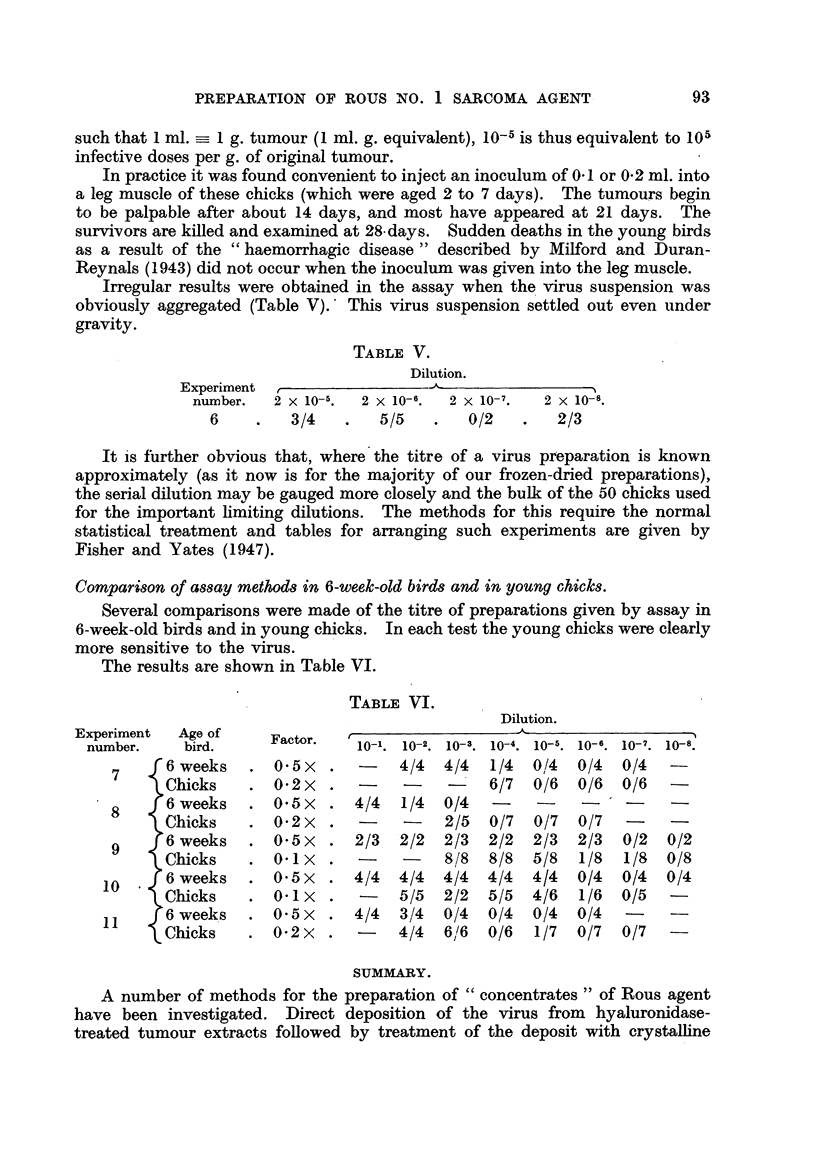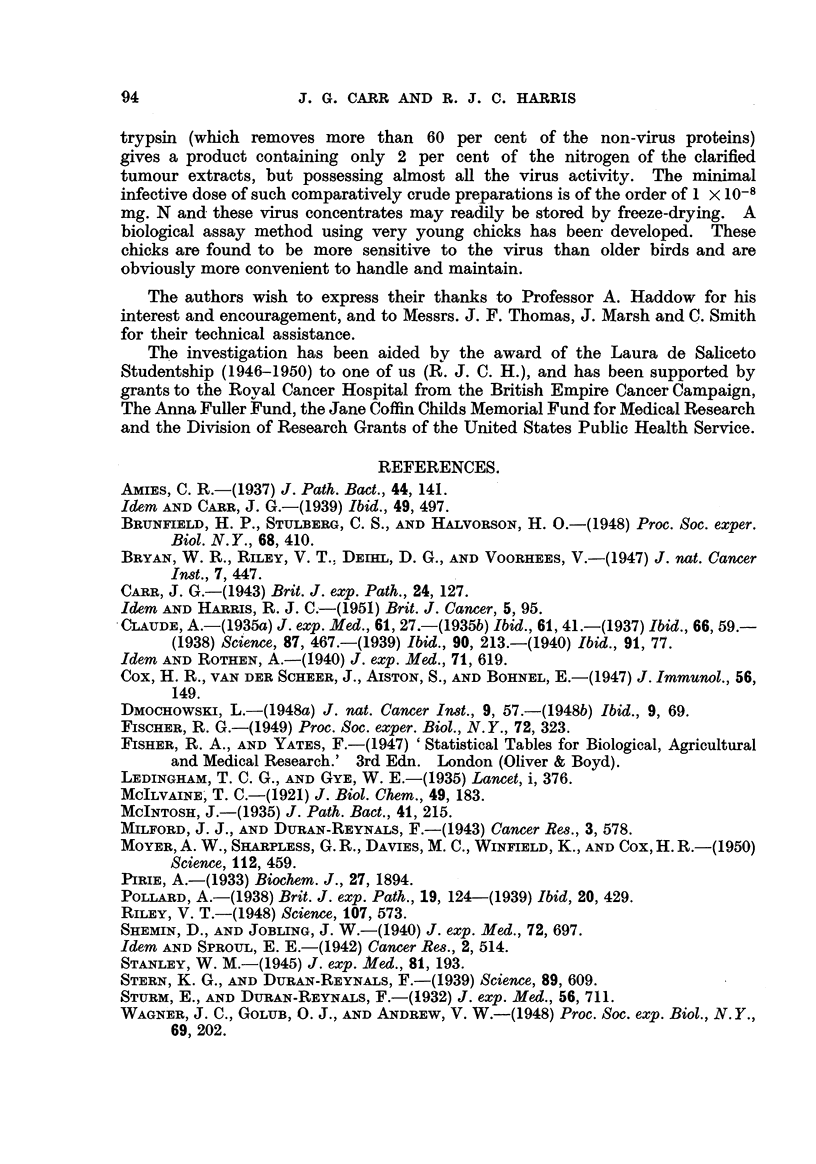# The Preparation and Assay of the Rous No. 1 Sarcoma Agent

**DOI:** 10.1038/bjc.1951.8

**Published:** 1951-03

**Authors:** J. G. Carr, R. J. C. Harris


### THE PREPARATION AND ASSAY OF THE ROUS No. 1 SARCOMA AGENT.

J. G. CARR AND R. J. C. HARRIS.*

*From the Chester Beatty Research Institute, Royal Cancer Hospital, London, S.W.3.*

Received for publication January 11, 1951.

THE major requirement for the investigation of the physical and chemical properties of the Rous No. 1 sarcoma agent is a source of " purified " agent of a uniformly high infectivity, and which may be stored without loss of this activity, since it is not always convenient to compress the investigation to within the relatively short period in which the purified agent is stable *in vitro*. This paper describes a method for the production of concentrates of the virus in a form suitable for storage, and the storage problem itself is considered in the following paper.

A variety of chemical and physical methods have hitherto been used for separating and concentrating the agent from biologically-inactive tumour protein and nucleoproteins.

Claude (1935*a*) found that such contaminants could be removed by adsorption on to freshly prepared aluminium hydroxide gel, and the carbohydrate contaminant by precipitation with gelatin. The agent was not directly affected, and a combined chemical fractionation and dialysis gave a 25-fold concentration in terms of dry weight (Claude, 1935*b*).

This method was also investigated by Dmochowski (1948*b*), who recorded a 50 per cent increase in the activity of the agent compared with the starting filtrate.

Shemin, Sproul and Jobling (1940), and Shemin and Sproul (1942) precipitated the agent from filtrates by addition of papain or of histone. The papain-agent complex (containing 50 to 70 per cent papain) was found to be at least as active as the original filtrate. The papain could be removed by fractional centrifugation after dissociation of the complex in salt solution. Amounts of agent corresponding to as little as $7 \times 10^{-7}$ mg. nitrogen (the range was $1{\cdot}3 \times 10^{-3}$ mg. to $7 \times 10^{-7}$ mg.) were found to be infective.

Concentration of the agent by centrifugation was first described by Ledingham and Gye in 1935 and fractional centrifugation methods have since been widely used (McIntosh, 1935 ; Amies, 1937 ; Claude, 1937, 1938, 1939, 1940 ; Claude and Rothen, 1940 ; Pollard, 1938, 1939 ; Stern and Duran-Reynals, 1939 ; Dmochowski, 1948*a*). Claude (1938, 1939) isolated a fraction corresponding to less than 3·5 per cent of the dry weight of the original extract which, in amounts corresponding to $4{\cdot}0 \times 10^{-10}$ mg. N, produced tumours in 13 to 17 days after subcutaneous inoculation into fowls. There are two difficulties in the separation and concentration of the agent by fractional centrifugation : (*a*) the viscosity of the initial tumour extract—which contains a mucoprotein, and (*b*) the tendency of the agent to aggregate, and to adsorb to tissue components.

* Junior Research Fellow, British Empire Cancer Campaign.

Pollard (1939) and Amies and Carr (1939) were able to overcome the first difficulty by precipitating the agent from a clarified extract by adjusting the pH to 5·0 to 5·5. The isoelectric point of the purified agent is 3·5, but tumour extracts deposit a mucoprotein at pH 5·0, which takes down the agent with it. The aggregated agent, etc., was then further concentrated by a low-speed centrifugation procedure. The aggregates were broken down by tryptic digestion at pH 8·8 to 9·0 and the released agent further " purified " by fractional centrifugation.

Alternatively, the mucoprotein may be hydrolysed by incubation of the extract with hyaluronidase, which has no deleterious effect upon the agent (Sturm and Duran-Reynals, 1932; Shemin and Sproul, 1942). The agent may then be deposited directly from the non-viscous extract.

Bryan, Riley, Deihl and Voorhees (1947) have avoided this difficulty by the simple, but uneconomical, procedure of rejecting the first, viscous extract.

A further method which has been claimed to apply to the purification of small amounts of agent is that of chromatography on Celite (Riley, 1948). The agent is strongly adsorbed to Celite from physiological salt solution, but may be eluted with very dilute (0·001 M) salt solution. In terms of the ratio, biological activity : nitrogen content, a 3- to 7-fold enrichment was obtained from a partially-purified tumour extract. Three of the above methods, papain precipitation, fractional centrifugation after precipitation at pH 5·0 and direct deposition after treatment of the initial extract with hyaluronidase, have been reinvestigated from the point of view of the preparation of large quantities of agent in a form suitable for storage. A further method, methanol precipitation, hitherto applied to a number of other animal viruses, has also been investigated.

Cox, van der Scheer, Aiston and Bohnel (1947) found that influenza virus (from allantoic fluid) could be precipitated in active form from suspensions in phosphate buffer at pH 7·0 to 8·0 by addition of methanol to a final concentration of 15 to 30 per cent. The method was applied to psittacosis virus purification by Wagner, Golub and Andrew (1948) and to mouse poliomyelitis by Brunfield, Stulberg and Halvorson (1948). Fischer (1949) found, however, that rabbit papilloma virus was reduced in titre after such precipitation and Moyer, Sharpless, Davies, Winfield and Cox (1950) have recently stressed the necessity for using 0·3 M buffer solutions for the extraction of influenza virus from the methanol precipitates.

### MATERIALS AND METHODS.

*Tumour.*

The Rous No. 1 tumour used was grown throughout in 6-week-old Brown Leghorn fowls from Dr. Greenwood's Edinburgh flock. Only fast-growing tumours, 20 to 30 days old were used. Such tumours are known to contain similar amounts of virus with little or no associated inhibitory material.

*1 : 10,000 HCN.*—One ml. M KCN was added to 8 ml. water and neutralized to phenol red with N HCl; 1 ml. added to 1 l. buffer gives a final concentration of 1:10,000.

*Buffers.*—In the pH range 2·2 to 9·0, McIlvaine's (1921) phosphate-citric acid buffer has been used. The buffer components are 0·2 M $Na_2HPO_4$ and 0·1 M citric acid. Of each buffer 50 ml. was diluted before use to 1 l. (0·005 M buffer).

*Lemco broth.*—Used as supplied by Baird & Tatlock.

*Trypsin.*—Crystalline trypsin (Armour Laboratories) containing 50 per cent magnesium sulphate.

*Hyaluronidase.*—Fresh rat testes were homogenized (Waring " blendor ") with 10 vol. water. The suspension was roughly filtered through surgical gauze and frozen-dried in 10 ml. aliquots in 25 ml. McCartney bottles. The contents of each bottle were reconstituted before use by the addition of 10 ml. water.

*Papain.*—British Drug Houses, Ltd.

*Centrifuges.*

The Rous agent becomes progressively more thermolabile as purification proceeds. Centrifuge B was used in a cold-room at 0° C., and C was adjusted to a chamber temperature of 0° C. The Sharples centrifuge (A) was cooled with running tap water only. The centrifugal procedures used are listed below and will be referred to in the rest of the paper by such abbreviations as (A, i).

*A. Sharples air-driven supercentrifuge, stainless steel bowls.*—(i) Clarification; clarifier bowl; maximum flow rate at 25,000 r.p.m. (ii) Deposition at pH 5·0 : Same bowl and conditions as (i). (iii) Deposition at pH 7·0. The same bowl as (i) and (ii), but 30 ml./minute flow at 48,000 r.p.m.

*B. Serval angle centrifuge, Type SS.*1, *lusteroid tubes.*—(i) Clarification; 5000 r.p.m. (3000 g.) for 20 minutes. (ii) Deposition at pH 7·0; 11,000 r.p.m. (14,400 g.) for 55 minutes.

*C. International refrigerated centrifuge, Type PR*-1.—High speed attachment and angle head No. 296; lusteroid tubes. (i) Clarification; 5000 r.p.m. (2000 g.) for 18 minutes. (ii) Deposition; 15,000 r.p.m. (17,000 g.) for 57 minutes.

*Waring blendor efficiency.*

Doubt has frequently been expressed, usually without supporting evidence, concerning the use of the Waring " blendor " for the maceration of tissues for the liberation of enzymes and viruses. It would obviously be tiresome to " mince " half-kilogramme batches of tumour with the " conventional " scissors and accordingly the efficiency of the Waring " blendor " was tested in the following way.

Tumours from two fowls were removed and small samples, totalling about 5 g., taken. The bulk of the tumour was extracted in the Waring " blendor " with 10 volumes of water containing 1:10,000 HCN, and the small sample " minced with scissors " and extracted into a similar medium. Suitable dilutions of these extracts were injected into fowls. The Waring " blendor " extract gave tumours at a dilution corresponding to $10^{-5}$ g. of tumour, while the other failed to give tumours at an equivalent of $10^{-4}$ g.

### *I. Precipitation with Papain.*

In the Waring " blendor " 44 g. fresh tumour tissue was disintegrated with 440 ml. 0·005 M buffer at pH 7·2 containing hyaluronidase and 1:10,000 HCN. The tissue suspension was clarified (B, i) and the agent deposited from the supernatant (B, ii). The combined pellets were resuspended in 25 ml. water by gentle pipetting and the suspension clarified (B, i). Aliquots of 1 ml. of the supernate were added to (*a*) 5 ml. water (control), (*b*) 5 ml. papain solution (Shemin and Jobling, 1940; Shemin and Sproul, 1942) and (*c*) 5 ml. 0·1 per cent trypsin (B.D.H.). The mixtures were then incubated at 37° C. for 1 hour. Tube (*b*)

developed a precipitate which was deposited. The control suspension (*a*) was assayed comparatively against (*b*)—resuspended papain-agent complex, etc., (*b*) —supernatant and (*c*). The assay showed that (*b*) deposit and (*c*) were devoid of any activity, (*b*) supernatant was weakly active and (*a*) control was highly active at $1{\cdot}0 \times 10^{-3}$. In view of the results obtained by Pirie in 1933, the inactivation by crude commercial trypsin was not unexpected, but the result with (*b*) was.

### *II. Precipitation with Methanol.*

(1) A fresh tumour tissue suspension was prepared by disintegration in the Waring " blendor " with 10 vol. of water containing 1:10,000 HCN. This suspension was clarified (A, i). The supernatant was then cooled to — 12° C. and methanolic buffer solutions prepared, and cooled to — 5° C. These buffers contained (*a*) 40 per cent methanol, (*b*) 50 per cent methanol and (*c*) 60 per cent methanol respectively in 0·005 M buffer. Equal volumes of (*a*), (*b*), (*c*) or buffer (*d*)-control, were added to volumes of the supernatant. The four groups were stored at 0° to 2° C. for 2 hours and then clarified (B, i). The supernatants (Sa, etc.) were set aside for assay and the deposits resuspended in an equal volume of 0·005 M buffer and re-clarified. These supernatants (Saa, etc.) were also assayed. Virus activity was retained in Sd, Sa and Sc. Activity was also found in Saa and, doubtfully, in Sbb ; Scc had no activity.

It was concluded that the precipitation with methanol was incomplete even at a concentration of 30 per cent and that methanol concentrations greater than 25 per cent tended to attenuate any precipitated agent.

(2) Method 1 was followed for the preparation of the initial clarified tumour extract. The control virus suspension was prepared by deposition from the clarified extract at pH 5·0 (A, ii), followed by resuspension of the deposit at pH 7·0. An equal volume of 50 per cent methanolic buffer was added under the same conditions as Method 1 and the resuspended deposit assayed against the extract. The assay showed that the alcohol-deposited agent had only 1 per cent of the activity of the control suspension.

(3) A similar clarified extract was prepared, cooled to 0° C. and the conditions of Method 2 again followed. Larger volumes were used and the alcohol-precipitated agent was deposited at low speed (A, i). The deposit was resuspended in 0·005 M buffer at pH 7·2, clarified (B, i) and the agent in the supernatant deposited (B, ii). The pellet was finally resuspended in 0·005 M buffer, pH 7·2, adjusted in concentration to the equivalent of 1 g. tumour tissue/ml. (10°—see Biological Assay) and assayed against the extract, similarly adjusted. The control contained $>$10,000 minimal infective doses per ml., whereas the alcohol deposited agent contained $<$1000 m.i.d. per ml.

### *III. Deposition after Precipitation at pH* 5·0.

A 10 per cent fresh tumour tissue suspension was prepared in the usual way, but with the omission of hyaluronidase. The suspension was clarified (A, i) and deposited (A, ii) after adjustment to pH 5·0 by addition of an equal volume of 0·005 M buffer at pH 5·0.

*Fractional centrifugation.*

The deposit from (A, ii) was resuspended in 0·005 M buffer at pH 7·0 (v/w of original tumour tissue taken) and clarified (B, i). The supernatant, S (Fig. 1)

was decanted and the deposit D, re-extracted with a similar volume of the same buffer and re-clarified (B, i) giving a deposit $D_3$ and a supernatant, $S_3$.

The agent, etc., of supernatant S was deposited (B, ii). The supernatant ($S_1$) was rejected and the agent deposit ($D_1$) re-extracted with 0·005 M buffer at pH 5·0 (25 per cent v/w original weight tumour) and re-clarified (B, i). This supernatant ($S_2$) was adjusted to pH 7·0, and deposit ($D_2$) resuspended in the same volume of 0·005 M buffer at pH 7·0. $D_2$, $S_2$ and $S_3$ were assayed in the usual way. $D_2$ contained more than 10,000 m.i.d./ml.; $S_3$, not more than 1000 and $S_2$ not more than 100 m.i.d./ml. (Fig. 1).

FIG. 1.

These results show that this method, although used successfully by Pollard (1939) and by Amies and Carr (1939), had certain limitations. With an initial volume of tumour extract of 5 litres (from 500 g. tumour) the volumes of suspension to be centrifuged at the pH 5 deposition stage were very large. One of the major difficulties with the Rous agent is its tendency to aggregate and procedures which attempt to isolate it by aggregation ought, therefore, to be avoided. The loss by aggregation is well illustrated in Fig. 1, since $S_3 \simeq D_2$ in titre (where $S_3$ derives from the second extract of the material aggregated at pH 5·0 for deposition). Moreover, deposit $D_1$ lost agent after *extraction* with 0·005 M buffer at pH 5·0 ($S_2$ assayed at $\ngtr 10^{-2}$) and the efficiency of the initial deposition process was thereby called into question.

The main reason why direct deposition of the agent from a clarified extract has not hitherto been attempted *on a large scale* is simply that suitable centrifuges have not been available. The air-driven Sharples supercentrifuge with a maximum speed of rotation of 50,000 r.p.m. (62,000 g.) was used by Stanley in 1945 for the concentration of influenza virus for vaccine production. Under such conditions the Rous agent may be deposited from a continuously-flowing non-viscous (i.e. hyaluronidase-treated) extract.

### *IV. Direct Deposition with the Sharples Centrifuge.*

1. *Boundary phenomenon.*

In the Waring " blendor " 145 g. fresh tumour tissue was disintegrated with 10 volumes of 0·005 M phosphate buffer at pH 7·2 containing hyaluronidase and

1:10,000 HCN. The suspension was clarified (A, i) and the virus deposited from the supernatant (A, iii). For deposition of the virus the stainless- steel centrifuge bowl was invariably lined with cellophane (7/1000 in.) which may (*a*) be readily withdrawn and which (*b*) minimizes contact of the virus with metals.

When unrolled this " liner " invariably showed a boundary, the distance of which from the bottom of the bowl, varied with the rate of flow of the virus suspension through the centrifuge. In terms of total nitrogen distribution, the deposit was uniformly distributed above and below the boundary, although the virus content of the top and bottom fractions differed considerably. The " liner " was therefore divided at the boundary and the deposits removed and separately resuspended in 0·01 M buffer at pH 7·2 (DB and DT). A few mg. per cent crystalline trypsin was added to each, the suspensions incubated at room temperature for 40 minutes and the virus re-deposited (A, iii) (SB, ST-supernatants and $DB_1$, $DT_1$-deposits). The separate fractions were analysed for total nitrogen. In this second deposition procedure neither fraction gave a boundary indicating that the material below the initial boundary was largely trypsin-digestible.

The results may be tabulated.

TABLE I.

| Fraction. | N, mg. | N, per cent total N. | Comparative assay. |
|---|---|---|---|
| *Bottom.* | | | |
| DB | 49·5 | 51·5 | — |
| SB | 43·2 | 45·0 | — |
| $DB_1$ | 6·3 | 6·5 | $10^{-2}$ |
| *Top.* | | | |
| DT | 47·0 | 48·5 | — |
| ST | 21·4 | 22·2 | — |
| $DT_1$ | 25·5 | 26·4 | $10^{-3}$ |

Trypsin digestion removed 87·5 per cent of the nitrogen of the bottom fraction, but only 45·5 per cent of that of the top fraction. Overall 67 per cent of the total nitrogen was thus removed. The virus concentrates ($DB_1 + DT_1$) contained 31·8 mg. nitrogen (33 per cent of the total nitrogen) of which 80 per cent (25·5 mg.) was recovered from the top fraction. The fractions $DB_1$ and $DT_1$ were assayed comparatively at $10^{-1}$, $10^{-2}$ and $10^{-3}$, and the figures recorded do not refer to the m.i.d.

2. *Nitrogen distribution after trypsin treatment.*

In the Waring " blendor " 520 g. fresh tumour tissue was disintegrated with 10 volumes of 0·005 M phosphate buffer at pH 7·2 containing 1:10,000 HCN—but without hyaluronidase—and the suspension clarified (A, i). Four litres of extract was obtained which was then treated with clarified (C, i) rat testes extract and to 2 l. of which was added *c.* 0·5 mg. per cent crystalline trypsin. The non-trypsinized half (X) was deposited (A, iii), the " liner " removed and divided at the boundary. The top (XT) fraction was resuspended in 0·005 M buffer, pH 7·2 and clarified (B, i). The supernatant from this (XTS) was assayed in the usual way.

The trypsinized half (Y) was first re-clarified (A, i) to remove a slight deposit which had formed, and the agent then deposited (A, iii). No boundary was found on the " liner " and the total deposit (YD) was resuspended in buffer and clarified (B, i). The supernatant (YDS) was assayed. Fractions were analysed for total nitrogen (Table II).

TABLE II.

| Fraction. | N, mg. | N, per cent total N. |
|---|---|---|
| X (no trypsin) | 1310 | 100 |
| Total deposit on liner | 208 | 16 |
| XTS | 13·8 | 1·05 |
| Y (trypsin) | 1310 | 100 |
| Total deposit on liner | 81·5 | 6·2 |
| YDS | 22·2 | 1·7 |

The virus-containing fractions, XTS and YDS, contained a total of 36 mg. nitrogen (1·375 per cent total nitrogen in original clarified tumour extract).

Assays showed that the trypsin-treated fraction (YDS) not only contained more nitrogen than the non-trypsinized XTS, but that, at a dilution of $10^{-6}$, YDS was more active than XTS. The trypsin removed again some 61 per cent of the nitrogen of the treated fraction.

3. *Efficiency of fractional centrifugation.*

A " liner " deposit was prepared from 110 g. fresh tumour tissue in the usual manner (p. 88).

The deposit ($D_1$) was resuspended in 100 ml., 0·005 M buffer, pH 7·0, and treated with crystalline trypsin for 1 hour at room temperature. (*a*) The suspension was then clarified (B, i), the deposit ($D_2$) discarded and (*b*) the virus deposited (B, ii) from the decanted supernatant ($S_1$). The supernatant ($S_2$) from this deposition was discarded and the deposit ($D_3$) resuspended in 60 ml. buffer and (*c*) re-clarified (C, i). This deposit ($D_4$) was rejected and virus again (*d*) deposited (C, ii) from the supernatant ($S_3$). The supernatant ($S_4$) from this deposition was assayed. The deposit ($D_5$) was resuspended in 30 ml. buffer and (*e*) re-clarified (C, i). The deposit ($D_6$) was negligible. The supernatant ($S_5$) was assayed in the usual way.

Results :

$S_5$ contained more than $10^7$ m.i.d. per ml.
$S_4$ contained not more than 10 m.i.d. per ml.
(Tumours in 2 out of 4 fowls at dilution $10^{-1}$.)

The separation is shown schematically in Fig. 2.

The results show that, although virus losses may be expected to occur by aggregation and removal in clarification procedures (into deposits such as $D_2$ and $D_4$) no loss occurs in the deposition stages, such as (*d*), since the amount of residual virus in $S_4$ was infinitesimal.

4. *Standard procedure.*

This procedure has been adopted for routine preparation of a virus concentrate suitable for preservation by freeze-drying.

Fig. 2.

A " liner " deposit was prepared from 375 g. fresh tumour tissue by the method described on p. 88.

The " liner " was removed and the total deposit ($D_1$) resuspended in 375 ml. 0·005 M phosphate buffer at pH 7·5 to 8·0 containing a few mg. per cent crystalline trypsin. The suspension was incubated at room temperature for 1 hour, clarified (B, i) giving a deposit $D_2$ (rejected) and a supernatant ($S_2$) from which the virus was deposited (B, iii). The supernatant ($S_3$) from this deposition was rejected and the deposit ($D_3$) resuspended in 300 ml. Lemco broth, distributed into McCartney bottles in 10 ml. aliquots and frozen-dried. Each fraction was assayed for protein nitrogen (insoluble in cold, 5 per cent trichloracetic acid) and total nitrogen.

The results are given in Table III.

Table III.

| Fraction. | Protein N, mg. | Non-protein N, mg. | Protein N as per cent total N. |
|---|---|---|---|
| Clarified tumour extract | 1760 | 464 | 79 |
| $D_1$ | 308 | 14 | 14 |
| $D_1$ (trypsinized) | 260 | 62 | 11·7 |
| $S_2$ | 184 | 62 | 8·25 |
| $D_2$ | 76 | — | 3·4 |
| $S_3$ | 140 | 62 | 6·3 |
| $D_3$ | 44 | — | 2·0 |

The results show that 44 mg. of the 308 mg. protein-nitrogen of the original Sharples deposit appeared as " virus concentrate " ($D_3$). Of the remaining 264 mg., 48 mg. appeared as acid-soluble nitrogen after trypsinization, 76 mg. was discarded as aggregated material ($D_2$) and 140 mg. was not sedimented under conditions in which the virus was deposited (i.e. this material was partially degraded by the trypsin). In this experiment 188 mg. N out of 308 mg. was removed as a result of the enzyme treatment. This amount (61 per cent) is in good agreement with the results of IV, 1 (67 per cent) and IV, 2 (61 per cent).

### DISCUSSION.

The results of the first three methods investigated show that in our hands neither papain precipitation nor methanol precipitation gave encouraging evidence of the concentration of active agent. Deposition by pH adjustment to pH 5·0 gave material with a high titre but had two major disadvantages in that large volumes of suspension had to be handled, and the agent, once aggregated, was very difficult to redisperse.

Investigation of the direct deposition of the agent by high-speed continuous flow centrifugation showed that the bulk of the heavy protein-containing material which sedimented more rapidly could be hydrolysed by short treatment with purified (crystalline) trypsin at room temperature at pH 8·0 to 9·0. Over 60 per cent of the nitrogen of the total deposit was rendered non-sedimentable by this treatment and the activity of the agent was apparently unimpaired. Virus " concentrates " thus prepared contained some 1·7 to 2·0 per cent of the nitrogen of the original extract and had titres greater than 1,000,000 m.i.d. per ml./g. tumour.

Such comparatively " unpurified " material as YDS (Table II) had an m.i.d. of less than $1 \times 10^{-8}$ mg. N—ten times as good, in fact, as the material obtained by Shemin and Jobling (1940) by papain precipitation.

It is difficult to express the " purity " of a virus preparation except in terms of a large number of factors, but the " concentrates " obtained by the above methods have been freed of more than 98 per cent of non-virus nitrogen, may readily be stored (Carr and Harris, 1951), recovered and subjected to further procedures designed to increase the physical, chemical and biological homogeneity of the agent.

The physical and chemical behaviour of the agent will form the substance of a later communication.

### *Biological assay.*

The various methods which have been used for the determination of the tumour-producing activity of Rous agent preparations fall essentially into two groups. In the first group serial dilutions are made and injected into fowls. The highest dilution at which a tumour is produced gives a " minimal infective dose " (m.i.d.). In the second group, the virus suspension of unknown titre is inoculated into a fowl, together with a standard virus suspension of known titre. The titres are compared either by the relative sizes of the tumours ultimately produced or by the time elapsing before detectable tumours arise.

This second group of methods, although useful, no doubt, in certain types of work, was avoided by us. There was no *a priori* reason for assuming that a constant standard could be obtained or that the subsequent comparison would be unaffected by such factors as the season of the year or the nature of the medium in which the virus was suspended. The first group (" limiting dilution " methods) had, also, the advantage of giving some degree of absolute measure corresponding to the actual number of tumour-producing particles.

Two injection routines have been favoured by previous workers ; either the intradermal or the intramuscular. The former allows several different dilutions to be tested in the same bird, but accurate localization of the injection is rendered difficult by the paper-thin structure of the avian skin. Moreover, the frequent occurrence of resistant fowls necessitates replication of the tests. Intramuscular injections involve a more readily standardized reaction site, but 6-week-old birds frequently show a variable and unpredictable resistance to the virus.

Such resistant birds may not react to 1000 times the virus dose which will produce tumours in susceptible birds, and if the end-point (m.i.d.) is dependent, as may be shown, upon a Poisson distribution of virus particles, then a large number of 6-week-old fowls would be required for an accurate assay.

It is inconvenient to use large numbers of birds of this age for a single assay and for such reasons the use of embryos or of day-old chicks, which may readily be obtained in large numbers, was investigated. Embryos were found to be less convenient than day-old chicks, since a tumour is only produced in the embryo, when (and where) a deliberate injury is inflicted at the time of inoculation (i.e. pock-count methods cannot be used). Moreover, the separate injection of each embryo is a laborious task.

The day-old chick is known to show less virus resistance than older birds (Carr, 1943). Such chicks may be used in large numbers, but, since metastasis to the site of injury produced by a non-infective inoculation may occur, only one test injection in each chick is possible (Carr, 1943).

Preliminary tests on serial decimal dilutions in groups of 50 chicks were encouraging. False negatives in birds receiving high concentrations of virus did not occur, indicating that the chicks were not manifesting resistance to the virus. The nature of the " tailing-off " of activity in the high dilutions indicated that an accurate estimate of the titre could be obtained. Table IV gives examples of assays from a few of the early tests.

TABLE IV.

| Experiment number. | Dilution. $1 \times 10^{-3}$. | $1 \times 10^{-4}$. | $1 \times 10^{-5}$. | $1 \times 10^{-6}$. | $1 \times 10^{-7}$. | $1 \times 10^{-8}$. |
|---|---|---|---|---|---|---|
| 1 | — | 3/3 | 4/4 | 4/5 | 0/5 | 0/5 |
| 2 | — | 4/4 | 5/5 | 6/6 | 1/7 | 0/7 |
| 3 | — | — | 6/6 | 2/6 | 1/5 | 0/6 |
| 4 | 8/8 | 3/7 | 1/7 | 0/7 | 0/7 | 0/7 |
| 5 | — | — | 6/6 | 6/6 | 1/7 | 0/7 |

In the dilution columns of these Tables (IV, V and VI) the numerator of the fraction shown refers to the number of tumours and the denominator to the number of inoculations. All titres are referred back to the wet weight of tumour tissue used initially. Thus $10^{\circ}$ is a concentration of virus product in suspension such that 1 ml. ≡ 1 g. tumour (1 ml. g. equivalent), $10^{-5}$ is thus equivalent to $10^5$ infective doses per g. of original tumour.

In practice it was found convenient to inject an inoculum of 0·1 or 0·2 ml. into a leg muscle of these chicks (which were aged 2 to 7 days). The tumours begin to be palpable after about 14 days, and most have appeared at 21 days. The survivors are killed and examined at 28 days. Sudden deaths in the young birds as a result of the "haemorrhagic disease" described by Milford and Duran-Reynals (1943) did not occur when the inoculum was given into the leg muscle.

Irregular results were obtained in the assay when the virus suspension was obviously aggregated (Table V). This virus suspension settled out even under gravity.

TABLE V.

| Experiment number. | Dilution. $2 \times 10^{-5}$. | $2 \times 10^{-6}$. | $2 \times 10^{-7}$. | $2 \times 10^{-8}$. |
|---|---|---|---|---|
| 6 | 3/4 | 5/5 | 0/2 | 2/3 |

It is further obvious that, where the titre of a virus preparation is known approximately (as it now is for the majority of our frozen-dried preparations), the serial dilution may be gauged more closely and the bulk of the 50 chicks used for the important limiting dilutions. The methods for this require the normal statistical treatment and tables for arranging such experiments are given by Fisher and Yates (1947).

*Comparison of assay methods in 6-week-old birds and in young chicks.*

Several comparisons were made of the titre of preparations given by assay in 6-week-old birds and in young chicks. In each test the young chicks were clearly more sensitive to the virus.

The results are shown in Table VI.

TABLE VI.

| Experiment number. | Age of bird. | Factor. | Dilution. $10^{-1}$. | $10^{-2}$. | $10^{-3}$. | $10^{-4}$. | $10^{-5}$. | $10^{-6}$. | $10^{-7}$. | $10^{-8}$. |
|---|---|---|---|---|---|---|---|---|---|---|
| 7 | 6 weeks | 0·5× | — | 4/4 | 4/4 | 1/4 | 0/4 | 0/4 | 0/4 | — |
| | Chicks | 0·2× | — | — | — | 6/7 | 0/6 | 0/6 | 0/6 | — |
| 8 | 6 weeks | 0·5× | 4/4 | 1/4 | 0/4 | — | — | — | — | — |
| | Chicks | 0·2× | — | — | 2/5 | 0/7 | 0/7 | 0/7 | — | — |
| 9 | 6 weeks | 0·5× | 2/3 | 2/2 | 2/3 | 2/2 | 2/3 | 2/3 | 0/2 | 0/2 |
| | Chicks | 0·1× | — | — | 8/8 | 8/8 | 5/8 | 1/8 | 1/8 | 0/8 |
| 10 | 6 weeks | 0·5× | 4/4 | 4/4 | 4/4 | 4/4 | 4/4 | 0/4 | 0/4 | 0/4 |
| | Chicks | 0·1× | — | 5/5 | 2/2 | 5/5 | 4/6 | 1/6 | 0/5 | — |
| 11 | 6 weeks | 0·5× | 4/4 | 3/4 | 0/4 | 0/4 | 0/4 | 0/4 | — | — |
| | Chicks | 0·2× | — | 4/4 | 6/6 | 0/6 | 1/7 | 0/7 | 0/7 | — |

SUMMARY.

A number of methods for the preparation of "concentrates" of Rous agent have been investigated. Direct deposition of the virus from hyaluronidase-treated tumour extracts followed by treatment of the deposit with crystalline trypsin (which removes more than 60 per cent of the non-virus proteins) gives a product containing only 2 per cent of the nitrogen of the clarified tumour extracts, but possessing almost all the virus activity. The minimal infective dose of such comparatively crude preparations is of the order of $1 \times 10^{-8}$ mg. N and these virus concentrates may readily be stored by freeze-drying. A biological assay method using very young chicks has been developed. These chicks are found to be more sensitive to the virus than older birds and are obviously more convenient to handle and maintain.

The authors wish to express their thanks to Professor A. Haddow for his interest and encouragement, and to Messrs. J. F. Thomas, J. Marsh and C. Smith for their technical assistance.

The investigation has been aided by the award of the Laura de Saliceto Studentship (1946–1950) to one of us (R. J. C. H.), and has been supported by grants to the Royal Cancer Hospital from the British Empire Cancer Campaign, The Anna Fuller Fund, the Jane Coffin Childs Memorial Fund for Medical Research and the Division of Research Grants of the United States Public Health Service.

### REFERENCES.

AMIES, C. R.—(1937) *J. Path. Bact.*, **44**, 141.

*Idem* AND CARR, J. G.—(1939) *Ibid.*, **49**, 497.

BRUNFIELD, H. P., STULBERG, C. S., AND HALVORSON, H. O.—(1948) *Proc. Soc. exper. Biol. N.Y.*, **68**, 410.

BRYAN, W. R., RILEY, V. T., DEIHL, D. G., AND VOORHEES, V.—(1947) *J. nat. Cancer Inst.*, **7**, 447.

CARR, J. G.—(1943) *Brit. J. exp. Path.*, **24**, 127.

*Idem* AND HARRIS, R. J. C.—(1951) *Brit. J. Cancer*, **5**, 95.

CLAUDE, A.—(1935*a*) *J. exp. Med.*, **61**, 27.—(1935*b*) *Ibid.*, **61**, 41.—(1937) *Ibid.*, **66**, 59.—(1938) *Science*, **87**, 467.—(1939) *Ibid.*, **90**, 213.—(1940) *Ibid.*, **91**, 77.

*Idem* AND ROTHEN, A.—(1940) *J. exp. Med.*, **71**, 619.

COX, H. R., VAN DER SCHEER, J., AISTON, S., AND BOHNEL, E.—(1947) *J. Immunol.*, **56**, 149.

DMOCHOWSKI, L.—(1948*a*) *J. nat. Cancer Inst.*, **9**, 57.—(1948*b*) *Ibid.*, **9**, 69.

FISCHER, R. G.—(1949) *Proc. Soc. exper. Biol., N.Y.*, **72**, 323.

FISHER, R. A., AND YATES, F.—(1947) 'Statistical Tables for Biological, Agricultural and Medical Research.' 3rd Edn. London (Oliver & Boyd).

LEDINGHAM, T. C. G., AND GYE, W. E.—(1935) *Lancet*, i, 376.

MCILVAINE, T. C.—(1921) *J. Biol. Chem.*, **49**, 183.

MCINTOSH, J.—(1935) *J. Path. Bact.*, **41**, 215.

MILFORD, J. J., AND DURAN-REYNALS, F.—(1943) *Cancer Res.*, **3**, 578.

MOYER, A. W., SHARPLESS, G. R., DAVIES, M. C., WINFIELD, K., AND COX, H. R.—(1950) *Science*, **112**, 459.

PIRIE, A.—(1933) *Biochem. J.*, **27**, 1894.

POLLARD, A.—(1938) *Brit. J. exp. Path.*, **19**, 124—(1939) *Ibid*, **20**, 429.

RILEY, V. T.—(1948) *Science*, **107**, 573.

SHEMIN, D., AND JOBLING, J. W.—(1940) *J. exp. Med.*, **72**, 697.

*Idem* AND SPROUL, E. E.—(1942) *Cancer Res.*, **2**, 514.

STANLEY, W. M.—(1945) *J. exp. Med.*, **81**, 193.

STERN, K. G., AND DURAN-REYNALS, F.—(1939) *Science*, **89**, 609.

STURM, E., AND DURAN-REYNALS, F.—(1932) *J. exp. Med.*, **56**, 711.

WAGNER, J. C., GOLUB, O. J., AND ANDREW, V. W.—(1948) *Proc. Soc. exp. Biol., N.Y.*, **69**, 202.